# Solid Pseudopapillary Neoplasm of the Pancreas: Clinicopathologic Feature, Risk Factors of Malignancy, and Survival Analysis of 53 Cases from a Single Center

**DOI:** 10.1155/2017/5465261

**Published:** 2017-09-28

**Authors:** He Song, Ming Dong, Jianping Zhou, Weiwei Sheng, Banghua Zhong, Wei Gao

**Affiliations:** Department of Gastrointestinal Surgery, The First Hospital of China Medical University, Shenyang 110001, China

## Abstract

**Introduction:**

Solid pseudopapillary neoplasm (SPN) of the pancreas is a rare tumor of low malignant potential. The aim of this study was designed to evaluate the clinicopathologic feature, predictive factors of malignancy, and survival from experience of a single center.

**Methods:**

53 consecutive patients who underwent surgery for a pathologically definitive SPN were retrospectively reviewed.

**Results:**

A total of 53 cases included 7 male cases and 46 female cases with the median age of 35.4 years (14–67). Abdominal pain and mass were the most common clinical presentations. The radiological presentations were consistent with solid and cystic pattern in 18 cases, solid pattern in 25 cases, and cystic pattern in 10 cases. The predominant location of tumor was pancreatic body and tail. The mean size of the tumors was 6.4 cm. Aggressive en bloc resection combined with organ-preserving should be indicated whenever feasible. Follow-up information was available for 48 patients with a median follow-up time of 48 months. The 5-year disease-specific survival was 95.7%. Incomplete capsule was not only the predictive factor of malignancy but also the significant predictor of disease-specific survival.

**Conclusion:**

Incomplete capsule may suggest a malignant SPN and a prognostic indicator of disease-specific survival. We recommend that surgeons consider a more radical resection with an incomplete capsule of tumor.

## 1. Introduction

Solid pseudopapillary neoplasm (SPN) is a rare pancreatic tumor predominantly affecting young women with low malignant potential [1] and was first reported by Frantz in 1959 [2]. It usually has a favorable prognosis, with just over 95% of patients reported as being disease-free after surgical resection and with less than 2% mortality [3]. The aim of this study was designed to evaluate the clinicopathologic feature, predictive factors of malignancy, and survival from 13-year experience of a single center.

## 2. Materials and Methods

Between January 2004 to January 2017, 53 consecutive patients with a definitive pathological diagnosis of SPN at the Department of General Surgery, The First Hospital of China Medical University, were retrospectively reviewed. Patients' demographic, clinical presentation, radiological details, surgical data, pathological features, long-term survival, and other relevant data were extracted from hospital records and evaluated. Outpatient records combined with telephone interviews were used for follow-up. The criteria of SPN were defined as malignant if it demonstrated extrapancreatic invasion, distant metastases, pancreatic parenchymal invasion, peripancreatic fat tissue infiltration, lymph node involvement, capsular invasion, or perineural or vascular invasion [4–7]. Patient status was due to the time of last follow-up as follows: no evidence of disease, alive with disease, dead of disease, surgical mortality, and dead of other causes. Survival time was defined as starting from the date of first operation until end of follow-up due to either death or end of data collection. Only “dead of disease” was considered an event in the analysis of disease-specific survival. Descriptive statistics were represented as mean ± standard deviation [SD] or as the median (range) or as proportion. The characteristics of the 2 patient groups (with benign and malignant SPNs) were compared. Univariable analysis was performed using *χ*^2^ test for categorical variables and Student's *t*-test for continuous variables. The Kaplan-Meier method was used to estimate survival. The log-rank test was used to analyze differences in patient survival. *P* value of less than 0.05 was considered significant. All statistical analyses were performed with the Statistical Package for Social Sciences (SPSS) 16.0 for Windows (Chicago, Illinois).

## 3. Results

### 3.1. Common Information

A total of 53 cases included 7 male cases and 46 female cases and the ratio of male/female was 1 : 6.6. Meanwhile the youngest is 14-year-old and the eldest is 67-year-old and the average age was 35.4 ± 13.4 years. The average of BMI was 23.7 ± 2.4 (18.6 to 28.5). There were 8 patients infected with Hepatitis B virus (HBV) and 2 patients infected with Hepatitis C virus (HCV).

### 3.2. Clinical Manifestations

The clinical presentation was upper abdominal pain in 20 cases (37.7%), abdominal distention in 9 cases (20.0%), abdominal mass in 16 cases (30.2%), incidental detection in 21 cases (39.6%), nausea and vomiting in 6 cases (11.3%), back pain in 3 cases (5.7%), and hematuria in 1 case (1.9%). No one presented the obstructive jaundice. The symptoms were nonspecific, and coexistence of two or more symptoms was often found.

### 3.3. Preoperative Examination

The level of tumor markers, including AFP, CEA, CA199, CA125, and CA242, was slightly increased in 6 patients, but none were diagnosed as malignant SPN. In addition, according to our hospital cases, the most preoperative laboratory tests were within normal limits.

The three most common forms of abdominal imaging were CT, ultrasound, and MRI, and accounted for 50 patients (94.3%), 48 patients (90.6%), and 10 patients (18.9%), respectively. The radiological appearance of SPN is typically characterized by well-encapsulated, heterogenous (solid and cystic) mass, whose solid component was enhanced on the arterial and venous phase but lower than normal pancreatic tissue. The radiological presentations were consistent with solid and cystic pattern in 18 cases, solid pattern in 25 cases, and cystic pattern in 10 cases. Calcification in the tumor and dilated main pancreatic duct were reported for 15 patients and 3 patients. There were 45 cases completely encapsulated and 8 cases incompletely encapsulated in the radiological image. 7 cases of incomplete capsule were malignant. Preoperative percutaneous biopsies and endoscopic ultrasound-fine needle aspiration (EUS-FNA) were performed in 2 cases and 3 cases. Among these cases, 3 cases were diagnosed as SPN, 1 case was diagnosed as dubious pancreatic tumor tissue, and another 1 case was misdiagnosed as pancreatic islet cell tumor.

15 cases (28.3%) were distributed on the pancreatic head, 32 cases (60.4%) were distributed on the pancreatic body and tail, 5 cases (9.4%) were distributed on the pancreatic neck, and 1 case (1.8%) was on the extrapancreatic site (located at site surrounded by the left kidney, spleen, and pancreatic tail). The mean size of the tumors was 6.4 ± 3.5 cm (2–14 cm).

### 3.4. Surgery

All the 52 cases had *R*_0_ resection. 6 cases underwent pancreaticoduodenectomy, 3 cases underwent pylorus-preserving pancreaticoduodenectomy, 3 cases underwent duodenum-preserving pancreatic head resection, 2 cases underwent cases of middle pancreatectomy, 15 cases underwent distal pancreatectomy with preserving-spleen, 13 cases underwent distal pancreatectomy with splenectomy, and 10 cases underwent enucleation. In 1 case of gastric involvement, another case of renal involvement, and 2 cases of transverse colon involvement, we performed tumor resection plus subtotal gastrectomy, nephrectomy, and colectomy. One case had palliative operation (*R*_2_). Radical resection was impossible because massive superior mesenteric vein involvement made mass reduction and graft reconstruction unfeasible. Total surgical period was 3.9 ± 1.2 hours (1–8.5 hours). Blood transfusion was given to 3 patients in the volume of 2 units, 2 units, and 4 units, respectively. No postsurgical adjuvant therapy was administered to any patient.

There were no surgical mortalities. Postsurgical complications occurred in 14 patients. The most common complication was pancreatic fistula (9 cases), followed by intra-abdominal abscess (4 cases) and gastric fistula (1 case). These complications were all resolved by conservative therapy. Statistics all above was listed in Table 1.

### 3.5. Pathological and Immunohistochemical Characteristics

The typical gross appearance of SPN is well capsulated and demarcated from the pancreas, with a mixture of solid, cystic component in various proportions.

Microscopically, tumor cells arranged around fibrovascular stalk forming pseudopapillary pattern, focal areas of hemorrhage, and necrosis could usually be found. Immunohistochemical staining showed that alpha 1-antichymotrypsin (AACT), Vinmentin, alpha 1-Antitrypsin (AAT), Neuron-Specific Enolase (NSE), Progesterone Receptor (PR), Synaptophysin, and so forth appear to have positive expression mostly and the positive rates for them were 95.7% (45/47), 88.1% (37/42), 82.5% (33/40), 70% (28/40), 63.9% (23/36), and 55.3% (21/38), respectively, as is shown in Table 2. Immunohistochemical staining of Ki-67 was detected in 8 patients, 5 cases were expressed positive, and 4 cases of the noted 5 patients were confirmed to be malignant; Ki-67 immunoreactivity of 4 malignant cases was less than 1%.

### 3.6. Characteristics of Malignant SPN

53 cases were pathologically confirmed as SPN, 10 cases (18.9%) were diagnosed as malignant, as follows: vascular infiltration was identified in 1 patient, pancreatic parenchymal invasion concurrent with peripancreatic fat tissue infiltration was in 4 cases, adjacent organ invasion was in 4 cases, and perineural invasion was in 1 case. One case of pancreatic parenchyma invasion suffered from recurrence and underwent a second operation after 8 years of the first resection. None had lymph node metastasis. No tumors presented severe nuclear atypia or a high mitotic rate.  Table 3 compared and summarized the characteristics between patients with benign and malignant tumor.

### 3.7. Predictive Factors of Malignancy

**Figure 1:**
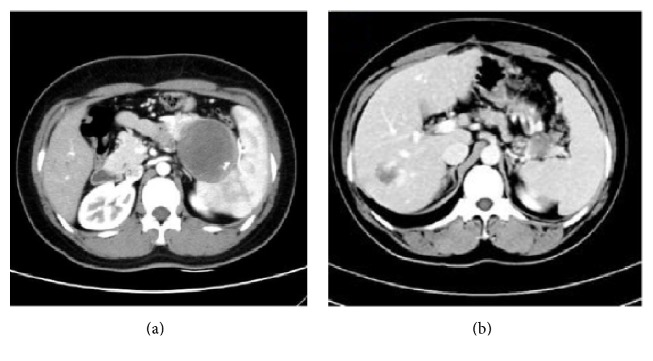
(a) The SPN locating at the tail of pancreas with complete capsule was confirmed to be benign. (b) The SPN locating at the tail of pancreas with incomplete capsule was confirmed to be malignant in the infiltrative growth pattern. Splenic vessels were invaded by the tumor. The mass at the right lobe of liver was confirmed to be hepatic hemangioma.

There is no statistical difference in the age, sex, symptom, serum tumor marker, tumor size, tumor location, calcification, and component of tumor. However, we found that incomplete capsule is significantly more in the malignant group (*P* < 0.001). The typical CT image in our cases series including complete and incomplete capsule was in Figures 1(a) and 1(b).

### 3.8. Follow-Up Results

**Figure 2:**
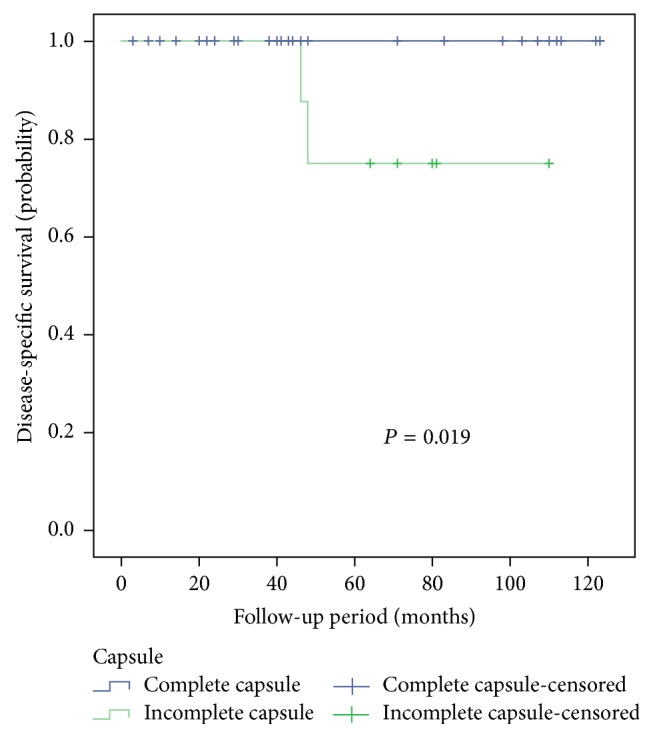
Cumulative Kaplan-Meier survival curve associated with capsule in SPN patients.

Follow-up data were collected by telephone or outpatient interview. Follow-up information was available for 48 patients with a median follow-up time of 48 months (3–123 months). 45 patients were alive at last follow-up, and 43 patients were without evidence of disease, including 3 patients after resection of adjacent organs. Two patients were alive with disease. One patient suffered from recurrence, and this patient who underwent enucleation developed tumor recurrence after 8 years and had a second operation. The other one who had the palliative operation survived well for 65 months. The disease-free survival was 91.7%. Three patients died: 2 died of SPN and 1 died of a traffic accident. Five patients were lost to follow-up. The 3-, 5-, and 10-year disease-specific survival were 100%, 95.7%, and 95.7%. By univariate analyses, incomplete capsule (*P* = 0.019) was significant predictor of disease-specific survival (Figure 2), but not age, sex, symptom, serum tumor marker, tumor size, tumor location, calcification, and component of tumor.

## 4. Discussion

SPN frequently occurs in young women. Law et al. [3] had performed a systematic review for English literature that indicated that female cases of SPN occupied 87.8% in the total of 2408 patients; the average age was 28.5 ± 13.7 years. In our study, female comprised 86.8% of total of 53 cases; meanwhile the average age of 53 cases was 35.4 ± 13.4 years. Although female patients with SPN outnumbered male patients, there were no gender-specific trends in expression of sex hormone receptor protein or clinicopathologic characteristics [8, 9]. Machado et al. [10] analyzed 34 cases including 7 male patients retrospectively and found that male patients with more aggressive behavior had distinct patterns of onset and aggressiveness compared to female patients. However, in our study, there was no correlation between malignancy and the age, sex, or BMI. The average of BMI of 53 cases was 23.7 ± 2.4 (18.6 to 28.5), so there was no connection between obesity and SPN. There were 8 patients infected with Hepatitis B virus (HBV) and 2 patients infected with Hepatitis C virus (HCV). Some study [11] reported that 62.5% of SPN patients were infected with HBV, which may correlate with the pathogenesis of SPN, but our study could not demonstrate this association.

The presentation of SPN is usually nonspecific. Similar to some study [1, 3], our data showed that abdominal pain was found in 37.7% of the patients and abdominal mass was seen in 30.2% of the patients. Many patients (39.6%) had no symptoms, and tumor was incidentally detected by imaging or medical examination. For most tumor size is big with the average diameter of 6.4 ± 3.5 cm; some patients presented symptoms of tumor compression affecting the alimentary tract, such as nausea and vomiting. However, there was no correlation between symptom and tumor size (*P* > 0.05). It is necessary to note that even if the tumor was located at the head of pancreas, no case caused obstructive jaundice due to the tumor exophytic growth way, which was consistent with some study [1, 3].

In the radiological image, SPN typically represented wide-ranging appearance from solid to cystic, a well capsulated mass, with solid and cystic component. In our study, concerning the solid component, the average of Hu value of plain CT scan, arterial phase, and venous phase was 37.8 Hu, 58.1 Hu, and 63.4 Hu. The Hu value between plain phase and arterial phase/venous phase was statistically significant (*P* < 0.05); however, the Hu value between arterial phase and venous phase was not (*P* > 0.05). Calcification may be present in some cases, whereas dilation of pancreatic duct is rare. MR imaging displaying intratumoral hemorrhage and the capsule of the SPN is better than CT [12]. But MRI data in our study was scarce. There was no correlation between malignancy and component of tumor and calcification in our study, consistent with [13]; however Hwang et al. regarded proportion of solid component was associated with malignancy [6]. Preoperative percutaneous biopsies and endoscopic ultrasound-fine needle aspiration (EUS-FNA) may establish an accurate preoperative diagnosis. Some studies [14, 15] had confirmed that EUS-FNA was a reliable tool that significantly increased diagnostic accuracy by characterizing the cytomorphological features. However, the procedure may cause tumor cell dissemination [16]. The 5 cases in our series did not experience any complications. There were 10 cases diagnosed malignant, and 7 of them were with incomplete capsule. Incomplete capsule defined a capsule that did not surround the entire periphery of the tumor in the radiological morphology. In the 7 tumors of incomplete capsule, there were 2 cases of exophytic growth pattern and 5 cases of infiltrative growth pattern. Most benign cases or cases with complete capsule exhibited exophytic pattern. Infiltrative growth pattern might cause the disruption of capsule and indicate the malignant behavior. Capsule morphology was significant between two groups (*P* < 0.05), consistent with some studies [17, 18]. Notably, two patients who died of SPN had the incomplete capsule with infiltrative growth pattern.

In laboratory tests, parameters were commonly within normal scope, so the routing laboratory parameters and tumor markers are of no help.

In our study, the predominant localization of tumor is the body and tail of the pancreas, followed by the head and the neck. It is worth mentioning that there was one case in the extrapancreatic sites, located at site surrounded by the left kidney, spleen, and pancreatic tail. This malignant tumor invaded the left kidney, caused the symptom of back pain and hematuria, and finally was resected with both tumor and kidney. The most common extrapancreatic sites were the mesocolon and ovary [19], sometimes even in the testicle [20]. It is cautious to explore carefully for ectopic pancreatic neoplasm to avoid misdiagnosis.

The preferred treatment of SPN is the surgery. Operational style depended on tumor's size, tumor's location, and intraoperative frozen section examination. Due to the low malignancy of tumor, organ-preserving operation should be performed whenever feasible [21]. For 3 cases of pylorus-preserving pancreaticoduodenectomy, it was found that tumor invaded pancreatic parenchyma or duodenum in the intraoperative frozen section examination, so pylorus-preserving pancreaticoduodenectomy was indicated. For 3 cases of duodenum-preserving pancreatic head resection, tumors were located at the head of pancreatic parenchyma, and it was unfeasible for enucleation because of damage of main pancreatic duct or superior mesenteric vessel. For the tumor on the pancreatic neck, 2 cases underwent central pancreatectomy. For the tumor on the pancreatic body and tail, distal pancreatic resection was carried out with splenectomy in 13 cases and with preserving-spleen in 15 cases. For the tumor's ectogenous growth tumor and complete amicula, away from the pancreatic duct and important pancreatic blood vessels, enucleation was carried out in 10 cases. One case had palliative operation (*R*_2_) because of massive superior mesenteric vein involvement. However, invasion to the portal vein or superior mesenteric artery should not be a contradiction of surgery [1]; some study carried out the vascular reconstruction with vein grafts after en bloc resection and had a favorable survival. For the patients whose tumor could not be completely resected, palliative surgery also provided a good survival. In addition, one case of SPN was found outside the pancreas, so careful exploration is particularly important in the operation.

Recurrence rate is estimated in 10–15% of patients after resection [22], whereas only 1 patient (1.9%) suffered from tumor recurrence in our study. The patient underwent the second enucleation during 96-month disease-free period during follow-up. Our series included 1 case of gastric involvement, another case of renal involvement, and 2 cases of transverse colon involvement. We performed tumor resection plus subtotal gastrectomy, nephrectomy, and colectomy resulting in good survival. Aggressive en bloc resection should be always be attempted including resection of concomitant metastases and infiltrative organ.

SPN rarely had lymph node involvement, no cases had lymph node involvement in our series, and extensive lymphatic dissection is not necessary.

Diagnosis of SPN mainly depends on the pathology and immunohistochemistry. In addition, our study showed the positive expression rates of Vinmentin, AACT, AAT, NSE, Synaptophysin, and PR were higher in immunohistochemistry. Ki-67 index has been suggested as a potential indicator of malignant potential and poor outcome of SPNs [23–25], but some study regarded the fact that it was not associated with malignancy [15]. The low Ki-67 index (≤5%) indicates a slow growth of the tumors. Ki-67 immunoreactivity proliferative index less than 1% of 4 malignant cases in our study had a good survival. The low proliferative index of Ki-67 may predict favorable outcome of malignant SPN. Regretfully, two patients died of SPN that were not detected by Ki-67.

We recommend that surgeons consider a more radical resection with an incomplete capsule of tumor. Papavramidis and Papavramidis [1] summarized the survival data in 467 patients and reported the 2-year and 5-year survival rate were 97% and 95%. Law et al. summarized the 1952 cases with recurrence and death of SPN, and 4.4% patients suffered recurrence; meanwhile 1.5% patients died of SPN. Similar to their findings, the 5-year disease-specific survival rate was 95.7% and recurrence rate was 1.9% in our study. Martin et al. [26] found that microscopic positive margin, invasion of surrounding structures, and size > 5 cm were not significant predictors of survival.

In summary, SPN is a rare pancreatic tumor, with a low-grade malignancy and strong female predilection. Clinical manifestations have no specificity, imaging examination is contributed by tumor location, and diagnosis relies on pathology. Surgery is the main method of treatment and the prognosis is good. Incomplete capsule may suggest a malignant SPN and a prognostic indicator of disease-specific survival.

## Figures and Tables

**Table 1:** Clinical characteristics of SPN patients.

Parameter	Patient number (*n* = 53)	%
*Age (year, average)*	35.4	
*Gender*		
Female	46	86.8%
Male	8	13.2%
*BMI (average)*	23.7	
*Hepatitis type*		
Hepatitis B virus	8	15.1%
Hepatitis C virus	2	3.8%
*Clinical presentation*		
Abdominal pain	20	37.7%
Abdominal distention	9	20.0%
Abdominal mass	16	30.2%
Incidental detection	21	39.6%
Nausea and vomiting	6	11.3%
Back pain	3	5.7%
Hematuria	1	1.9%
*Component of tumor*		
Solid and cystic	18	34.0%
Solid	25	47.2%
Cystic	10	18.9%
*Calcification*	15	28.3%
*Dilated main pancreatic duct*	3	5.7%
*Tumor location*		
Head	15	28.3%
Body	32	60.4%
Neck	5	9.4%
Extrapancreatic site	1	1.8%
*Tumor size (cm)*	6.4	
*Operation type*		
Pancreaticoduodenectomy	6	11.3%
Pylorus-preserving pancreaticoduodenectomy	3	5.7%
Duodenum-preserving pancreatic head resection	3	5.7%
Middle pancreatectomy	2	3.8%
Distal pancreatectomy with preserving-spleen	15	28.3%
Distal pancreatectomy with splenectomy	13	24.5%
Enucleation	10	18.9%
Palliative operation	1	1.9%
*Surgical period (hour average)*	3.9	
*Postsurgical complications*		
Pancreatic fistula	9	17.0%
Intra-abdominal abscess	4	7.5%
Gastric fistula	1	1.9%
*Capsule pattern*		
Complete capsule	45	84.9%
Incomplete capsule	8	15.1%
*Benign tumor*	43	81.1%
*Malignant tumor*	10	18.9%
Vascular infiltration	1	
Pancreatic parenchymal invasion	4	
Adjacent organ invasion	4	
Perineural invasion	1	

**Table 2:** Immunohistochemical staining.

Parameter	Positive	Total	%
AACT	45	47	95.7%
Vinmentin	37	42	88.1%
AAT	33	40	82.5%
NSE	28	40	70%
PR	23	36	63.9%
Synaptophysin	21	38	55.3%
Ki-67	5	8	62.5%

**Table 3:** Predictive factors of malignant SPN.

Factor	Benign	Malignant	*P* value
*Average age*	34.1 ± 12.0	41.0 ± 17.8	NS
*Average BMI*	23.6 ± 2.5	24.1 ± 1.9	NS
*Gender*			NS
Male	6	1	
Female	37	9	
*Symptoms*			NS
Present	27	4	
Absent	16	6	
*Serum tumor marker*			NS
Elevated	37	10	
Normal	6	0	
*Average tumor size (cm)*			NS
<5 cm	20	2	
>5 cm	23	8	
*Tumor location*			NS
Head	12	3	
Body and tail	27	5	
Neck	4	1	
Extrapancreatic site	0	1	
*Calcification condition*			NS
Calcification	11	4	
Noncalcification	32	6	
*Component of tumor*			NS
Solid and cystic	13	5	
Solid	19	4	
Cystic	11	1	
*Pattern of capsule*			*P* < 0.001
Complete capsule	42	3	
Incomplete capsule	1	7	

## References

[1] Papavramidis T., Papavramidis S. (2005). Solid pseudopapillary tumors of the pancreas: review of 718 patients reported in english literature. *Journal of the American College of Surgeons*.

[2] Frantz V.

[3] Law J. K., Ahmed A., Singh V. K. (2014). A systematic review of solid-pseudopapillary neoplasms: are these rare lesions?. *Pancreas*.

[4] Goh B. K. P., Tan Y.-M., Cheow P.-C. (2007). Solid pseudopapillary neoplasms of the pancreas: an updated experience. *Journal of Surgical Oncology*.

[5] Morikawa T., Onogawa T., Maeda S. (2013). Solid pseudopapillary neoplasms of the pancreas: an 18-year experience at a single Japanese Institution. *Surgery Today*.

[6] Hwang J., Kim D. Y., Kim S. C., Namgoong J.-M., Hong S.-M. (2014). Solid-pseudopapillary neoplasm of the pancreas in children: can we predict malignancy?. *Journal of Pediatric Surgery*.

[7] Kang C. M., Choi S. H., Kim S. C., Lee W. J., Choi D. W., Kim S. W. (2014). Predicting recurrence of pancreatic solid pseudopapillary tumors after surgical resection: a multicenter analysis in Korea. *Annals of Surgery*.

[8] Tien Y.-W., Ser K.-H., Hu R.-H., Lee C.-Y., Jeng Y.-M., Lee P.-H. (2005). Solid pseudopapillary neoplasms of the pancreas: is there a pathologic basis for the observed gender differences in incidence?. *Surgery*.

[9] Uppin S. G., Hui M., Thumma V. (2015). Solid-pseudopapillary neoplasm of the pancreas: a clinicopathological and immunohistochemical study of 33 cases from a single institution in Southern India. *Indian Journal of Pathology and Microbiology*.

[10] Machado M. C. C., Machado M. A. C., Bacchella T., Jukemura J., Almeida J. L., Cunha J. E. M. (2008). Solid pseudopapillary neoplasm of the pancreas: distinct patterns of onset, diagnosis, and prognosis for male versus female patients. *Surgery*.

[11] Sun G., Chen C., Yao J., Shi H., He Y., Z W. (2008). Diagnosis and treatment of solid pseudopapillary tumor of pancreas: a report of 8 cases with review of domestic literature. *Chinese Journal of General Surgery*.

[12] Yu M. H., Lee J. Y., Kim M. A. (2010). MR imaging features of small solid pseudopapillary tumors: retrospective differentiation from other small solid pancreatic tumors. *American Journal of Roentgenology*.

[13] Yin Q., Wang M., Wang C. (2012). Differentiation between benign and malignant solid pseudopapillary tumor of the pancreas by MDCT. *European Journal of Radiology*.

[14] Khashab M. A., Kim K., Lennon A. M. (2013). Should we do EUS/FNA on patients with pancreatic cysts? the incremental diagnostic yield of EUS over CT/MRI for prediction of cystic neoplasms. *Pancreas*.

[15] Hosokawa I., Shimizu H., Ohtsuka M. (2014). Preoperative diagnosis and surgical management for solid pseudopapillary neoplasm of the pancreas. *Journal of Hepato-Biliary-Pancreatic Sciences*.

[16] Fais P. O., Carricaburu E., Sarnacki S. (2009). Is laparoscopic management suitable for solid pseudo-papillary tumors of the pancreas?. *Pediatric Surgery International*.

[17] Guerrache Y., Soyer P., Dohan A. (2014). Solid-pseudopapillary tumor of the pancreas: MR imaging findings in 21 patients. *Clinical Imaging*.

[18] Chung Y. E., Kim M.-J., Choi J.-Y. (2009). Differentiation of benign and malignant solid pseudopapillary neoplasms of the pancreas. *Journal of Computer Assisted Tomography*.

[19] Cheuk W., Beavon I., Chui D. T. Y., Chan J. K. C. (2011). Extrapancreatic solid pseudopapillary neoplasm: report of a case of primary ovarian origin and review of the literature. *International Journal of Gynecological Pathology*.

[20] Michal M., Bulimbasic S., Coric M. (2016). Pancreatic analogue solid pseudopapillary neoplasm arising in the paratesticular location. The first case report. *Human Pathology*.

[21] Naar L., Spanomichou D.-A., Mastoraki A., Smyrniotis V., Arkadopoulos N. (2017). Solid pseudopapillary neoplasms of the pancreas: a surgical and genetic enigma. *World Journal of Surgery*.

[22] Geers C., Pierre M., Jean-François G. (2006). Solid and pseudopapillary tumor of the pancreas—review and new insights into pathogenesis. *American Journal of Surgical Pathology*.

[23] Serra S., Chetty R. (2008). Revision 2: an immunohistochemical approach and evaluation of solid pseudopapillary tumour of the pancreas. *Journal of Clinical Pathology*.

[24] Yang F., Jin C., Long J. (2009). Solid pseudopapillary tumor of the pancreas: a case series of 26 consecutive patients. *The American Journal of Surgery*.

[25] Watanabe Y., Okamoto K., Okada K., Aikawa M., Koyama I., Yamaguchi H. (2017). A case of aggressive solid pseudopapillary neoplasm: comparison of clinical and pathologic features with non-aggressive cases. *Pathology International*.

[26] Martin R. C. G., Klimstra D. S., Brennan M. F., Conlon K. C. (2002). Solid-pseudopapillary tumor of the pancreas: a surgical enigma?. *Annals of Surgical Oncology*.

